# Everolimus long-term use in patients with tuberous sclerosis complex: Four-year update of the EXIST-2 study

**DOI:** 10.1371/journal.pone.0180939

**Published:** 2017-08-09

**Authors:** John J. Bissler, J. Chris Kingswood, Elzbieta Radzikowska, Bernard A. Zonnenberg, Elena Belousova, Michael D. Frost, Matthias Sauter, Susanne Brakemeier, Petrus J. de Vries, Noah Berkowitz, Maurizio Voi, Severine Peyrard, Klemens Budde

**Affiliations:** 1 Department of Pediatric Nephrology, St. Jude Children’s Research Hospital, Le Bonheur Children’s Hospital, and the University of Tennessee Health Science Center, Memphis, Tennessee, United States of America; 2 Department of Nephrology, Royal Sussex County Hospital, Brighton, United Kingdom; 3 Department of Lung Diseases, National Tuberculosis and Lung Diseases Research Institute, Warsaw, Poland; 4 Department of Internal Medicine, Universitair Medisch Centrum, Utrecht, The Netherlands; 5 Department of Pediatrics, The Russian National Research Medical University named after N.I. Pirogov of the Ministry of Health of the Russian Federation, Moscow, Russia; 6 Department of Pediatric Neurology, Minnesota Epilepsy Group, St. Paul, Minnesota, United States of America; 7 Department of Nephrology, Medizinische Klinik und Poliklinik IV, Klinikum der Universität München, Munich, Germany; 8 Department of Nephrology, Charité Universitätsmedizin, Berlin, Germany; 9 Division of Child and Adolescent Psychiatry, University of Cape Town, Cape Town, South Africa; 10 Department of Oncology, Novartis Pharmaceuticals Corporation, East Hanover, New Jersey, United States of America; 11 Department of Oncology, Novartis Pharmaceuticals S.A.S., Rueil-Malmaison, France; Medizinische Universitat Graz, AUSTRIA

## Abstract

**Objectives:**

We examined the long-term effects of everolimus in patients with renal angiomyolipoma associated with tuberous sclerosis complex or sporadic lymphangioleiomyomatosis.

**Methods:**

Following favorable results from the double-blind core phase of EXIST-2 (NCT00790400), patients were allowed to receive open-label everolimus (extension phase). Patients initially randomly assigned to everolimus continued on the same dose; those who were receiving placebo crossed over to everolimus 10 mg/day. Dose modifications were based on tolerability. The primary end point was angiomyolipoma response rate, defined as a ≥50% reduction from baseline in the sum volume of target renal angiomyolipomas in the absence of new target angiomyolipomas, kidney volume increase of >20% from nadir, and angiomyolipoma-related bleeding grade ≥2. The key secondary end point was safety.

**Results:**

Of the 112 patients who received ≥1 dose of everolimus, 58% (95% CI, 48.3% to 67.3%) achieved angiomyolipoma response. Almost all patients (97%) experienced reduction in renal lesion volumes at some point during the study period. Median duration of everolimus exposure was 46.9 months. Sixteen (14.3%) patients experienced angiomyolipoma progression at some point in the study. No angiomyolipoma-related bleeding or nephrectomies were reported. One patient on everolimus underwent embolization for worsening right flank pain. Subependymal giant cell astrocytoma lesion response was achieved in 48% of patients and skin lesion response in 68% of patients. The most common adverse events suspected to be treatment-related were stomatitis (42%), hypercholesterolemia (30.4%), acne (25.9%), aphthous stomatitis and nasopharyngitis (each 21.4%). Ten (8.9%) patients withdrew because of an adverse event. Renal function remained stable, and the frequency of emergent adverse events generally decreased over time.

**Conclusions:**

Everolimus treatment remained safe and effective over approximately 4 years. The overall risk/benefit assessment supports the use of everolimus as a viable treatment option for angiomyolipoma associated with tuberous sclerosis complex or sporadic lymphangioleiomyomatosis.

**Trial registration:**

ClinicalTrials.gov NCT00790400

## Introduction

Tuberous sclerosis complex (TSC) is a genetic disorder with a birth incidence of 1 in 6000 and results in growth of hamartomas in organs such as the brain, kidneys, skin, lungs, eyes, and heart [1,2]. In the brain, subependymal giant cell astrocytomas (SEGAs) develop in up to 20% of patients and their growth can potentially lead to ventricular obstruction, acute hydrocephalus, and death [3,4]. Skin lesions of various types including hypopigemented macules, ungual fibromas, and facial angiofibromas are the most readily visible TSC manifestation with more than 90% of patients possessing one or more lesions [4].

Angiomyolipomas are the most common renal lesions associated with TSC, occurring in up to 80% of patients [5–8]. They are also seen in 33%–50% of patients with sporadic lymphangioleiomyomatosis (LAM) [9]. Angiomyolipomas usually manifest as multifocal, bilateral, asymptomatic disease, but as the lesions exceed 3 to 4 cm, they may become symptomatic [10–12] and carry an increased risk for aneurysms that can lead to hemorrhage [11,13]. Renal angiomyolipomas associated with TSC or sporadic LAM (sLAM) exhibit upregulated activity of mammalian target of rapamycin (mTOR) resulting from mutations in either *TSC1* or *TSC2* genes [4,14]. The TSC consensus guidelines recommend mTOR inhibitors as first-line therapy for asymptomatic growing angiomyolipomas measuring >3 cm in diameter, whereas selective embolization and kidney-sparing resection are acceptable second-line therapies [15]. The EXIST-2 (EXamining everolimus In a Study of Tuberous sclerosis complex) study demonstrated superiority of everolimus over placebo in angiomyolipoma response rate, time to angiomyolipoma progression, and skin lesion response rate in patients with angiomyolipoma associated with either TSC or sLAM [16,17]. Results from an interim analysis of the open-label extension phase demonstrated stability of everolimus effects over time [18]. Here we report data from the end of the EXIST-2 extension phase to evaluate the longer-term efficacy and safety of everolimus in treating renal angiomyolipoma.

## Methods

### Study participants

A complete description of study participants, design, and outcomes has been published [16,18]. In short, patients ≥18 years of age with a definitive diagnosis of TSC per consensus criteria or sLAM confirmed by biopsy or chest computed tomography (CT) with ≥1 angiomyolipoma measuring ≥3 cm on longest diameter using CT/magnetic resonance imaging (MRI) were eligible. Patients were excluded if their angiomyolipoma necessitated surgery at randomization or if they had angiomyolipoma-related bleeding or embolization within 6 months before randomization. Patients who had carbon monoxide diffusion capacity (DL_CO_) ≤35%, below-normal oxygen saturation at rest, or oxygen saturation ≤88% on a 6-minute walking test (6MWT, with up to 6 L/min of oxygen) were also excluded [16].

The study was reviewed by an independent ethics committee or institutional review board for each center (see S1 Table for list of committees/review boards), and was conducted in compliance with Good Clinical Practice and under the principles of the Declaration of Helsinki. Informed written consent was obtained from each patient or the patient’s guardian before randomization. The protocol of this trial and supporting CONSORT checklist are available as supporting information (see S1 Protocol and S1 CONSORT Checklist).

### Study design and treatment

EXIST-2 was a prospective, double-blind, randomized, parallel group, placebo-controlled, multicenter phase 3 study of everolimus 10 mg/day versus placebo [16,18]. During the double-blind core phase, patients were unblinded if they had any evidence of angiomyolipoma progression, and those receiving placebo were allowed to cross over to receive open-label everolimus. The study was unblinded on September 9, 2011, after a planned interim review showed everolimus to be highly effective and safe, while patients receiving placebo experienced continued deterioration [18]. An extension phase ensued in which patients who had been on everolimus during the core phase continued to receive the same dose and those who had been on placebo were switched to open-label everolimus (10 mg/day) (S1 Fig). Dose reductions to 5 mg/day or 5 mg/every other day were permitted based on safety. The extension phase continued until 4 years after the last patient was randomly assigned, ensuring patient follow-up of 4 to 5 years. Any patient who discontinued everolimus had a follow-up visit 28 days after the last dose to assess safety.

This analysis from EXIST-2 presents all data from patients who received everolimus during either the core or the extension phase of the study up to the data cutoff date of February 4, 2015.

### Study outcomes

The primary efficacy outcome was the angiomyolipoma response rate, defined as the proportion of patients with a confirmed angiomyolipoma response (≥50% reduction in the sum volume of target renal angiomyolipomas [≥1 cm in longest diameter] relative to baseline in the absence of new target angiomyolipomas, kidney volume increase of >20% from nadir [to account for possible growth in non-target lesions], and angiomyolipoma-related bleeding grade ≥2) [16]. Initial response required confirmation by second MRI. All MRI scans were analyzed in a central institute by independent radiologists. Additional end points included response duration, time to angiomyolipoma response and progression, proportion of patients with ≥30% reduction in angiomyolipoma volume, proportion of patients with angiomyolipoma-related surgery, skin lesion response rate (≥50% improvement on skin lesions, based on Physicians Global Assessment), SEGA response rate (≥50% reduction in sum volume of target SEGA lesions in the absence of new target SEGA lesion with no worsening of nontarget SEGA lesions and no new or worsening hydrocephalus). Pulmonary function tests were monitored in patients with LAM. Skin lesion types included in the assessment for skin lesion response were facial angiofibromas, shagreen patches, periungual or subungual fibromas, hypomelanotic macules, and forehead plaques.

Safety assessments included collection of all adverse events (AEs) and serious AEs, with their severity. AEs were assessed according to the National Cancer Institute Common Terminology Criteria for Adverse Events, version 3.0 [19]. Serious AEs were defined as those that were fatal or life-threatening, resulted in persistent or signifnicant disability/incapacity, constituted a congenital anomaly/birth defect, or required inpatient hospitalization or prolongation of existing hospitalization. Clinically notable AEs, defined as those AEs eliciting specific clinical interest in connection with everolimus, were also reported. Renal function was assessed using glomerular filtration rate (GFR), which was calculated using the Modification of Diet in Renal Disease formula [20]. Severe renal impairment was defined as a GFR <30 mL/min/1.73 m^2^ (chronic kidney disease stage ≥4).

### Statistical analysis

Combined data from the core and extension phases were analyzed for all patients who received at least one dose of everolimus using an intention-to-treat principle. Descriptive statistics were used to summarize baseline data and efficacy variables. The best percentage change from baseline at any time in the sum volume of target angiomyolipomas was represented by a waterfall plot. A piecewise linear mixed model with repeated measurements was used to link the post-baseline sum volume of angiomyolipoma lesions to the duration on treatment. The model included the sum volume of angiomyolipoma lesions at baseline as a covariate and considered two periods of time: the period in the first 3 months, and the period beyond the first 3 months. Kaplan-Meier curves were constructed for median duration of angiomyolipoma response. Summary statistics determined median time to angiomyolipoma response and progression, with statistics given as point estimates with 95% confidence interval (CI). Exact 95% CIs for angiomyolipoma, skin lesion, and SEGA responses were obtained from the Clopper-Pearson method. For median time to angiomyolipoma, skin lesion, and SEGA responses, 95% confidence interval were calculated with the LIFETEST procedure using the method of Brookmeyer & Crowley, 1982 [21]. Box plots depict GFR and creatinine values over time with mean, median, and interquartile range indicated. Statistical analyses were performed using SAS software (SAS Institute Inc., Cary, NC, USA).

## Results

### Baseline characteristics

**Fig 1:**
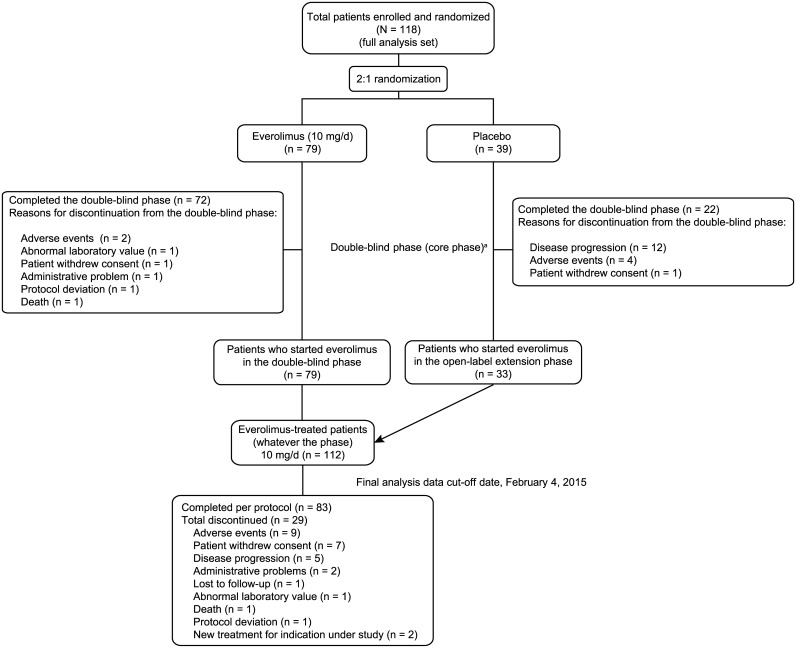
CONSORT flow diagram of patient disposition in the double-blind followed by open-label periods. ^a^Patients with angiomyolipoma progression were unblinded at the end of the double-blind phase, and patients who were on placebo were allowed to cross over to open-label everolimus.

Overall, 112 patients received at least one dose of everolimus and were included in this analysis: 79 patients originally randomly assigned to everolimus and 33 patients who switched to open-label everolimus from placebo. At the data cutoff (February 4, 2015), 83 patients (74.1%) completed the treatment duration as per protocol (Fig 1).

Most (65.2%) patients were women, and their median age was 32.2 years at baseline (Table 1). More than five target angiomyolipoma lesions at baseline were observed in 59.8% of patients, and 29.5% of patients had renal angiomyolipoma measuring ≥8 cm. The median sum of volumes of target angiomyolipoma lesions was 92.1 (range, 2.8–1611.5) cm^3^. More than one-third of patients (37.5%) had undergone angiomyolipoma-related surgery before initiating everolimus, and the median GFR at baseline was 86 (range, 23–178) mL/min/1.73 m^2^. Fifty patients (44.6%) had ≥1 target SEGA lesion.

**Table 1:** Patient baseline characteristics.

Characteristic	Everolimus N = 112
Age, median (range), years	32.2 (18.1–61.6)
Age category, years, n (%)	
<30	49 (43.8)
≥30	63 (56.3)
Sex, n (%)	
Male	39 (34.8)
Female	73 (65.2)
Race, n (%)	
White	99 (88.4)
Asian	11 (9.8)
Other^a^	2 (1.8)
Diagnosis of TSC,^b^ n (%)	107 (95.5)
Diagnosis of sporadic LAM, n (%)	5 (4.5)
Diagnosis of LAM, n (%)	24 (21.4)
Presence of ≥1 SEGA lesion, n (%)	50 (44.6)
Presence of ≥1 skin lesion, n (%)	107 (95.5)
Facial angiofibromas or forehead plaque	105 (93.8)
Ungual or periungual fibroma	68 (60.7)
Hypomelanotic macules (three or more)	69 (61.6)
Shagreen patch	54 (48.2)
'Confetti' skin lesions	30 (26.8)
Prior renal angiomyolipoma—related surgery, n (%)	42 (37.5)
Prior nephrectomy, n (%)	21 (18.8)
Bilateral renal angiomyolipoma lesions, n (%)	88 (78.6)
Longest diameter of largest renal angiomyolipoma lesion, n (%)	
≥8 cm	33 (29.5)
≥4 cm and <8 cm	64 (57.1)
≥3 cm and <4 cm	7 (6.3)
<3 cm	6 (5.4)
Unknown	0
Not applicable	2 (1.8)
Target renal angiomyolipoma lesions ≥1 cm, n (%)	
0	2 (1.8)
1–5	43 (38.4)
6–10	67 (59.8)
Sum of volumes of target renal angiomyolipoma lesions	
Number of patients with ≥1 target renal angiomyolipoma	110
Median (range), cm^3^	92.1 (2.8–1611.5)

Abbreviations: LAM = lymphangioleiomyomatosis; SEGA = subependymal giant cell astrocytoma; TSC = tuberous sclerosis complex.

^a^Designates patients who were of mixed race.

^b^All patients with diagnosed TSC had ≥2 major features of TSC. In patients with both LAM and renal angiomyolipoma, another feature must have been identified to assign TSC diagnosis.

### Everolimus exposure

The median time on study was 47.2 (range, 0.9–65.3) months, and the median duration of everolimus exposure was 46.9 (range, 0.5–63.9) months. The median dose intensity was 8.7 (range, 1.9–19.3) mg/day, and the relative dose intensity (ratio of administered dose to planned dose) was 0.86. Most (82.1%) patients were exposed to everolimus for ≥2.8 years, and 12.5% had exposure for ≥4.5 years. Ten (8.9%) patients discontinued the study because of AEs. Approximately 80% of patients required ≥1 dose interruption and/or reduction; AEs were the most common reason (66.1% for dose interruptions, 59.8% for dose reductions). At 4 years into the study, approximately 60% of patients were taking 10 mg/day of everolimus (as per protocol), approximately 30% of patients were taking between 5 and <10 mg/day of everolimus (mostly 5 mg/day) and 10% to 15% of patients were taking between 0 and <5 mg/day (mostly 5 mg/every other day) out of the 58 patients who had at least 4 years of exposure to everolimus in the study.

### Renal angiomyolipoma response

Of the 112 patients with ≥1 target renal angiomyolipoma at baseline, a confirmed response was achieved in 65 patients (58.0%; 95% CI, 48.3%–67.3%) at any time. The median time to angiomyolipoma response was 2.89 months (95% CI, 2.79 to 3.19). A best overall result of stable disease (between <50% reduction in lesion volume from baseline and <25% increase in volume over nadir) was reported in 34 patients (30.4%; 95% CI, 22.0%–39.8%); one patient (0.9%; 95% CI, 0.0%–4.9%), experienced disease progression within 18 weeks of the start of everolimus as a best response. Twelve (10.7%; 95% CI, 5.7%–18.0%) patients were not evaluable for response, primarily because of missing baseline kidney volume measurements.

**Fig 2:**
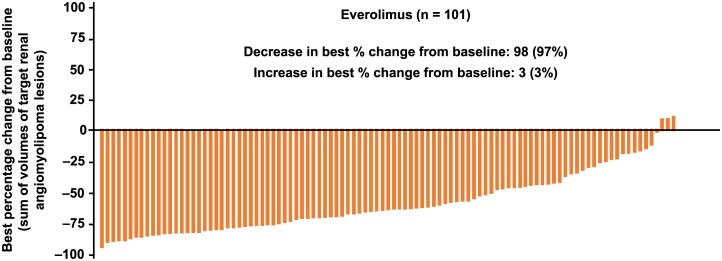
Best percentage reduction in the sum volume of target renal angiomyolipomas each individual patient reported at any time point in the study in 101 evaluable patients.^a^ ^**a**^11 patients were considered “non-evaluable” due to missing overall angiomyolipoma response status at each radiological assessment. Among the 12 patients with a best overall response with the status “not evaluable”, only one patient reported at least one radiological assessment with a non-missing overall angiomyolipoma response status.

**Fig 3:**
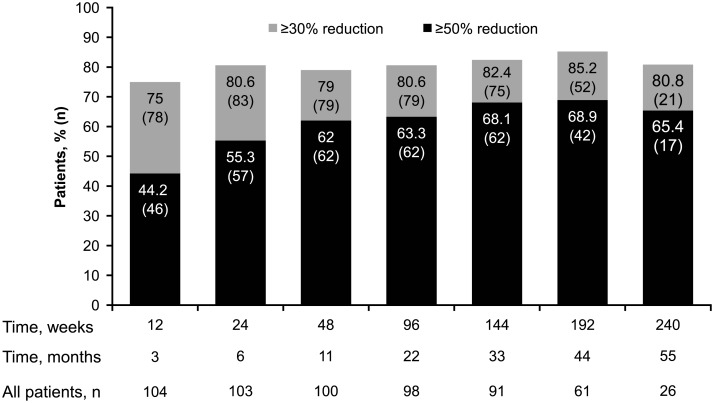
Renal angiomyolipoma response rate with everolimus over time.

**Fig 4:**
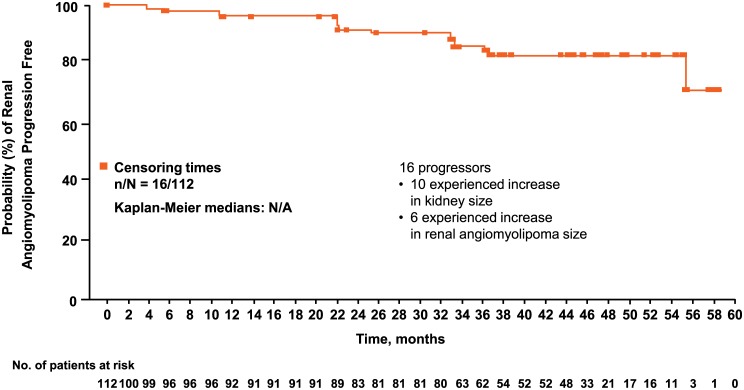
Time to renal angiomyolipoma progression.

At least one reduction from baseline in the sum of volumes of target angiomyolipoma lesions was observed in 98 (97%) of 101 evaluable patients at some point in the study (Fig 2). Renal angiomyolipoma volume reductions of ≥30% were maintained for ≥75% of patients at all time points. The proportion of patients achieving ≥50% reductions increased over time (Fig 3). The longitudinal analysis confirmed that longer treatment duration was associated with lower post-baseline sum volumes of angiomyolipoma lesions, especially within the first 3 months after treatment was initiated. During the initial 3-month period, a 46% (95% CI, 37%–54%) mean reduction in the post-baseline sum volumes of angiomyolipoma lesions was estimated. Beyond this period, three additional months of treatment resulted in a slight reduction of less than 2% (95% CI, <1%–2%) in the post-baseline sum volumes of angiomyolipoma lesions. Overall, renal angiomyolipoma progression was observed in 16 (14.3%; 95% CI, 8.4%–22.2%) patients (Fig 4). Reasons for progression were increased angiomyolipoma size (6 patients) and kidney enlargement (10 patients); 9 continued the treatment despite disease progression because of a perceived clinical benefit. Progressive disease was preceded by dose reduction or interruption in 13 of the 16 patients (81.3%), similar to the reduction/interruption rate in the overall study population (80.4%, 90/112). Only 2 of the 65 patients who achieved a confirmed angiomyolipoma response had disease progression based on their lowest angiomyolipoma volume during the course of the trial. The duration from the first response to angiomyolipoma progression or the last available radiologic assessment ranged from 3.0 to 55.5 months.

### Skin lesion response

Of the 107 patients with ≥1 skin lesion at baseline, investigators noted response in 73 patients (68.2%; 95% CI, 58.5%–76.9%; complete clinical response [n = 1, 0.9%; 95% CI, 0.0%–5.1%] and partial response of ≥50% to <100% [n = 72, 67.3%; 95% CI, 57.5%–76.0%]); 29 (27.1%; 95% CI, 19%–36.6%) patients had stable disease. The proportion of responders increased steadily over time (from 23% [24/103] at week 12 to 70% [39/56] at week 204). The median time to skin lesion response was 8.41 months (95% CI, 5.59–11.53). None of the 73 responders experienced progression of skin lesions.

### Subependymal giant cell astrocytoma lesion response

**Fig 5:**
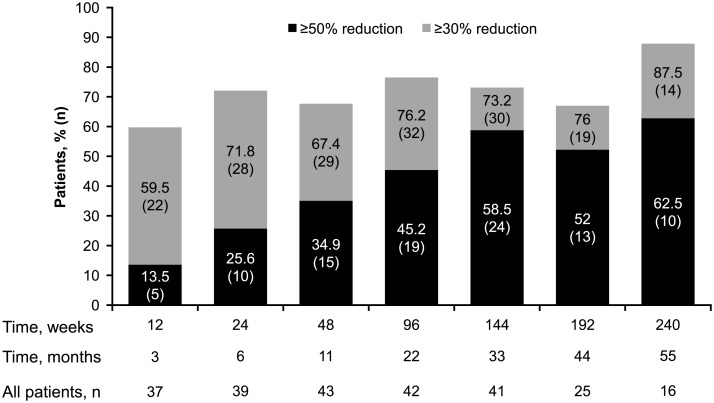
Reduction in SEGA volume with everolimus over time in patients with renal angiomyolipomas.

Among 50 patients with ≥1 baseline SEGA lesion measuring ≥1.0 cm, the response rate was 48% (24/50; 95% CI, 33.7%–62.6%), and 42% of patients (21/50; 95% CI, 28.2%-56.8%) achieved stable disease (five were not evaluable). Patients experienced gradual decreases in median SEGA volumes over time (week 12, –35.2%; week 144, –53.3%; and week 240, –54.4%). More than half the patients had ≥30% reduction in SEGA volume at all time points, and the proportion of patients with ≥50% reduction in SEGA volume increased over time (Fig 5). The median time to SEGA response was 8.31 months (95% CI, 5.55–11.30 months). None of the 24 SEGA responders experienced SEGA progression during the study.

### Changes in pulmonary function

Among 29 patients with LAM who were treated with everolimus, forced expiratory volume in 1 second (FEV_1_), forced vital capacity (FVC), and DL_CO_ decreased slightly over time. The median percentage change in FEV_1_ from baseline was –3.45% at week 24, –5.88% at week 96, and –9.00% at week 192. The median percentage change in FVC was 0% at week 24, –1.25% at week 96, and –4.29% at week 192. The median percentage change in DL_CO_ was –2.69% at week 24, –10.19% at week 96, and –10.62% at week 192. In addition, there was an increase in total lung capacity (median percentage change in TLC: week 24, 0%; week 96, 0.89%; week 192, 7.13%) and residual volume (median percentage change in RV: week 24, 0%; week 96, 3.94%; week 192, 14.29%) over time. Percentage changes and absolute values over time are provided in Table 2.

**Table 2:** Pulmonary function tests in patients with LAM over time.

	Everolimus n = 29		
	Week 24 n = 23	Week 96 n = 22	Week 192 n = 18
FEV_1_			
Baseline, median (min, max), L	2.8 (1.2, 3.6)	2.55 (1.2, 3.5)	2.8 (1.2, 3.6)
Assessment, median (min, max), L	2.6 (1.3, 3.6)	2.55 (1.0, 3.5)	2.45 (0.9, 3.4)
Median percentage change from baseline	–3.45	–5.88	–9
FVC			
Baseline, median (min, max), L	3.6 (1.8, 5.2)	3.6 (1.8, 5.2)	3.65 (2.1, 5.2)
Assessment, median (min, max), L	3.6 (2.0, 5.7)	3.6 (1.7, 5.4)	3.6 (2.1, 5.7)
Median percentage change from baseline	0	–1.25	–4.29
DL_CO_			
Baseline, median (min, max), mmol/min/Kpa	6.01 (2.7, 9.6)	5.76 (2.7, 9.6)	6.01 (2.7, 9.6)
Assessment, median (min, max), mmol/min/KPa	5.74 (1.3, 8.2)	5.23 (2.2, 9.4)	5.95 (2.4, 7.0)
Median percentage change from baseline	–2.69	–10.19	–10.62

Abbreviations: DL_CO_ = diffusing capacity of the lung for carbon monoxide; FEV_1_ = forced expiratory volume in 1 second; FVC = forced vital capacity.

### Safety outcomes

Most of the reported AEs were of grade 1 or 2 in severity. Individual grade 3 AEs occurred at a frequency ≤4.5%, and the most frequently reported that occurred in ≥2% were decreased blood phosphorus (laboratory abnormality; 4.5%), hypophosphatemia (per investigator; 2.7%), amenorrhea (3.6%), and epilepsy (2.7%). Individual grade 4 AEs were infrequent; the most common was blood uric acid increased (2.7%). The most common AEs suspected to be related to everolimus (≥20% of the patients) were stomatitis (42%), hypercholesterolemia (30.4%), acne (25.9%), and aphthous stomatitis and nasopharyngitis (21.4% each). Emergence of individual AEs was generally highest in the first year and decreased each subsequent year (Table 3). Serious AEs were observed in 37.5% of the patients, most frequently epilepsy (5.4%) and pneumonia (2.7%). Several AEs that have been previously noted with everolimus were monitored closely for their clinical impact. Infections were noted in 91.1%. Most infections involved the upper respiratory tract and were mild to moderate in severity. In addition to administration of appropriate non-drug therapy and concomitant medication, infections lead to everolimus dose interruptions or adjustments in 41 patients (36.6%). Stomatitis/related events were reported in 73.2% of patients. Hemorrhage-related events (prolonged activated partial thromboplastin time, decreased hemoglobin, menorrhagia, epistaxis, vaginal hemorrhage, international normalized ratio increased, and metrorrhagia) were reported in 49.1% of patients. Female fertility—related events (including secondary amenorrhea) occurred in 47.9% (34 of 71 females between 10 and 55 years of age) of patients, and 31% (22/71) experienced ≥1 episode of amenorrhea. Three patients reported grade 3 amenorrhea suspected to be related to everolimus. Two patients reported amenorrhea events requiring dose adjustment; none led to study discontinuation. Noninfectious pneumonitis occurred in two patients (both grade 2).

**Table 3:** Adverse events by preferred term regardless of relationship to study drug and by year of emergence (>15% of patients).

Adverse events, n (%)	≤12 months N = 112	13–24 months n = 101	25–36 months n = 100	37–48 months n = 91	49–60 months n = 52
Stomatitis	46 (41.1)	9 (8.9)	5 (5.0)	5 (5.5)	2 (3.8)
Nasopharyngitis	36 (32.1)	21 (20.8)	20 (20.0)	20 (22.0)	6 (11.5)
Acne	28 (25.0)	8 (7.9)	6 (6.0)	2 (2.2)	0
Headache	26 (23.2)	11 (10.9)	6 (6.0)	4 (4.4)	1 (1.9)
Hypercholesterolemia	25 (22.3)	13 (12.9)	11 (11.0)	7 (7.7)	1 (1.9)
Aphthous stomatitis	21 (18.8)	15 (14.9)	9 (9.0)	5 (5.5)	2 (3.8)
Fatigue	19 (17.0)	2 (2.0)	4 (4.0)	4 (4.4)	2 (3.8)
Cough	18 (16.1)	4 (4.0)	4 (4.0)	3 (3.3)	0
Diarrhoea	17 (15.2)	7 (6.9)	7 (7.0)	4 (4.4)	1 (1.9)
Mouth ulceration	17 (15.2)	6 (5.9)	5 (5.0)	2 (2.2)	0
Nausea	17 (15.2)	5 (5.0)	2 (2.0)	3 (3.3)	0

Note that an adverse event is counted only in the time period in which it started.

None of the treated patients reported angiomyolipoma-related bleeding during the study. The most common AEs requiring additional therapy included nasopharyngitis and stomatitis (33% each). One death (status epilepticus) was reported and was not suspected by the investigator to be treatment related.

### Renal function

**Fig 6:**
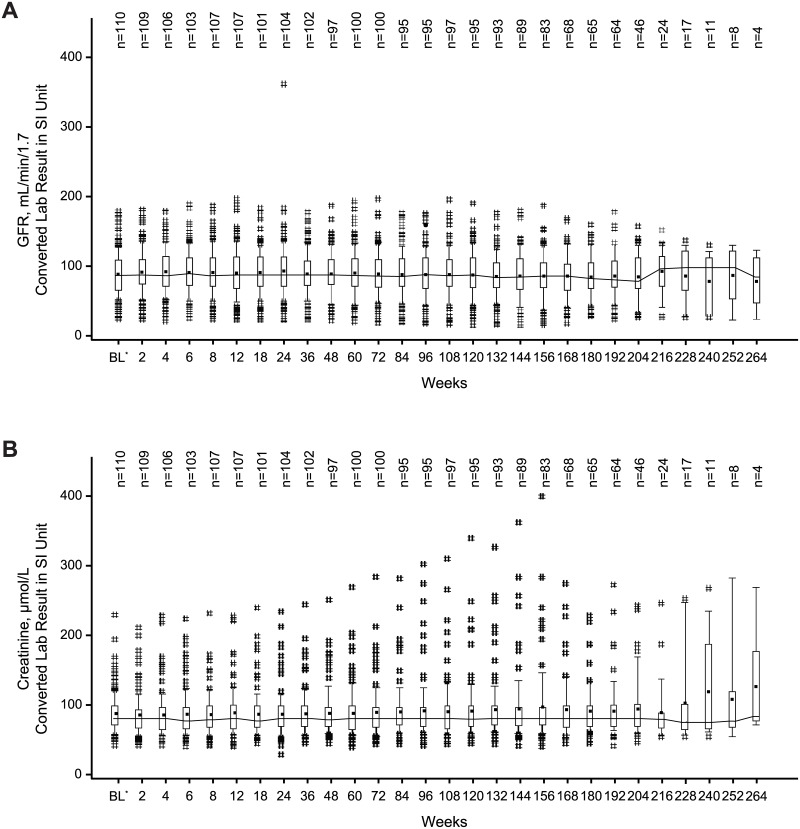
Median GFR (A) and creatinine (B) over time. Medians are connected by lines, means are displayed as dots. Boxes are drawn from P25 to P75. Whiskers extend from P10 to P90. # indicates values that lie outside [P10, P90]. *Baseline assessment is the last performed before start of everolimus. Post-baseline laboratory assessments performed at unplanned schedule or more than 28 days after discontinuation of everolimus are not presented. Only results from central laboratory are included. Abbreviations: BL = baseline; GFR = glomerular filtration rate; P = percentile.

Most patients had GFR ≥30 mL/min/1.73 m^2^ or normal serum creatinine values while receiving treatment with everolimus (92.9% and 83.9%, respectively). Median GFR and serum creatinine values remained stable during the everolimus treatment (Fig 6). Severe renal impairment (postbaseline GFR <30 mL/min/1.73 m^2^) was observed in eight (7.1%) patients. All eight patients had GFR <60 mL/min/1.73 m^2^ at baseline, including three patients with GFR <30 mL/min/1.73 m^2^ before everolimus initiation. In addition, 8 of 23 patients with GFR <60 mL/min/1.73 m^2^ at baseline attained a GFR below 30 mL/min/1.73 m^2^ while on everolimus, whereas in 15 patients GFR remained stable or increased. Grade 1 or 2 elevations in serum creatinine were observed in 15.2% of patients, and one patient (0.9%) had a grade 3 elevation in serum creatinine. Among the 18 patients with grade ≥1 elevations in serum creatinine, half reported normal creatinine level at baseline and half reported grade 1 or 2 serum creatinine increase before starting everolimus. Renal events occurred in 5% (4/79) of everolimus- and 15% (6/39) of placebo-treated patients during the double-blind period (median treatment duration, 8.8 and 7.8 months, respectively) [16,17] and in 20.5% of the 112 everolimus-treated patients for this final analysis (treatment duration, 46.9 months). Significant worsening of existing proteinuria (continuous urine dipstick ≥2 stages higher than baseline) occurred in two patients.

### Angiomyolipoma-related clinical events

Among the 112 patients who received everolimus, two patients underwent angiomyolipoma-related interventions. One patient receiving everolimus for 1.5 years underwent embolization after pain worsened in the right flank (progressive disease). Another patient underwent elective left partial nephrectomy following everolimus discontinuation (after 3 years of exposure) in an apparent attempt to use everolimus to reduce tumor burden prior to surgery.

## Discussion

This analysis from the conclusion of treatment in EXIST-2 supports the long-term efficacy and safety of everolimus in patients with renal angiomyolipoma associated with TSC or sLAM. Renal angiomyolipoma response continued to improve from 41.8% in the core phase (median exposure, 8.8 months) to 58% in the extension phase (median exposure, 46.9 months) [16]. Clinically relevant renal angiomyolipoma reductions persisted over time as shown by the longitudinal data analysis which demonstrated an important reduction in the first 3 months of treatment and a continuous but less marked reduction later on. Low rates of renal angiomyolipoma progression were observed.

Over the 4-year duration of everolimus treatment, angiomyolipoma-related complications or procedural interventions were uncommon, and no treated patient experienced an angiomyolipoma-associated hemorrhage, whereas one-third of the patients experienced major renal interventions before entering the trial.

Everolimus was also associated with sustained reductions in SEGA volume and improvement in skin lesions. In the subset of patients with TSC-associated LAM, a lower than expected rate of decline in pulmonary function was reported. A 12-month, placebo-controlled, double-blind study in patients with LAM reported an annual decline in FEV_1_ of 10% in patients taking placebo (*n* = 43) [22], whereas the current study reported a decline of 5.9% after nearly 2 years of everolimus treatment. Together, the evidence supports a multisystem clinical impact of everolimus on various manifestations of TSC.

The longer-term safety profile of everolimus was consistent with that previously reported in TSC-associated clinical settings with everolimus [23–26]. AEs occurred in most patients but were managed by dose adjustment in the majority. After the placebo arm was discontinued, it was difficult to distinguish between true everolimus side effects and AEs due to TSC (eg, infections were just as common in the placebo as in the everolimus group in the double-blind phase [16]). Some AEs, however, were predictable or known effects of mTOR inhibitors, often attributed to downregulation of cellular turnover (eg, amenorrhea, mouth ulcers). Nearly three-quarters of the patients completed this long-term study as per protocol. Stomatitis, an event known to be associated with everolimus, was the most frequent AE. While infections were common and mostly mild to moderate in severity during the study, it should be noted that severe life-threatening infections have been reported in patients treated with everolimus for TSC [27]. The frequency of emergent AEs of all types decreased over time. Renal function remained stable in most patients, and no angiomyolipoma-related bleeding events were reported. Immediate surgical intervention with embolization was performed in one patient (during the study period), and nephrectomy was performed in another (after treatment discontinuation). The observation of a single embolization while on everolimus over a median exposure of approximately 4 years indicates that treatment was successful and suggests a need for longer-term mTOR inhibition to continue to prevent or slow tumor regrowth, preserve renal function, and reduce the need for future embolization. This reduction in hemorrhage frequency could be due to restoration of a more appropriate mTORC1 axis in the TSC mutant vascular pericytes, thus preventing vascular remodeling and aneurysm formation [28]. Better angiographic data are required to assess the vascular effects on therapy [10]. Patients included in this study were at high risk for long-term renal deterioration because most of them had large, bilateral angiomyolipomas at baseline. Sustained clinical responses to everolimus in angiomyolipoma and other lesional manifestations of TSC—such as skin lesions, SEGA, and LAM—support everolimus as a viable treatment option for TSC and its potential to be a disease-modifying therapy in these patients.

Given the magnitude of effect with everolimus in the primary analysis, maintenance of an untreated arm was unethical, thus justifying the omission of the placebo arm in the extension phase. A planned noninterventional follow-up of EXIST-2 will provide longer-term data to determine angiomyolipoma characterizations for up to 1 year after the discontinuation of everolimus. Results of EXIST-2 demonstrate that everolimus is a safe and effective longer-term therapy for patients with renal angiomyolipoma associated with TSC or sLAM not requiring immediate surgical intervention. Early treatment with everolimus may prevent progressive damage to renal tissue, reduce renal-related complications and the need for renal intervention, and improve patient outcomes.

## Supporting information

S1 Table List of ethics committees and/or intuitional review boards that approved the EXIST-2 study. (DOCX)

S1 Protocol EXIST-2 study protocol. (PDF)

S1 CONSORT Checklist CONSORT 2010 checklist of information to include when reporting a randomized trial. (DOC)

S1 Fig Study design. CT = computed tomography; EIAED = enzyme-inducing antiepileptic drug; MRI = magnetic resonance imaging; sLAM = sporadic lymphangioleiomyomatosis; TSC = tuberous sclerosis complex. ^a^Enrollment occurred between April 28, 2009, and December 30, 2010. ^b^Dose adjusted based on toxicity. ^c^Angiomyolipoma progression by central review or occurrence of adverse event of angiomyolipoma-related bleeding grade 2 or worse.
(EPS)
